# *C*. *elegans*-based screen identifies lysosome-damaging alkaloids that induce STAT3-dependent lysosomal cell death

**DOI:** 10.1007/s13238-018-0520-0

**Published:** 2018-04-02

**Authors:** Yang Li, Yu Zhang, Qiwen Gan, Meng Xu, Xiao Ding, Guihua Tang, Jingjing Liang, Kai Liu, Xuezhao Liu, Xin Wang, Lingli Guo, Zhiyang Gao, Xiaojiang Hao, Chonglin Yang

**Affiliations:** 1 Department of Pharmacology, Key Laboratory of Metabolism and Molecular Medicine (The Ministry of Education), School of Basic Medical Science, Fudan University, Shanghai, 200032 China; 2 State Key Laboratory of Phytochemistry and Plant Resources in Western China, Kunming Institute of Botany, Chinese Academy of Sciences, Kunming, 650021 China; 3 State Key Laboratory of Molecular Developmental Biology, Institute of Genetics and Developmental Biology, Chinese Academy of Sciences, Beijing, 100101 China; 4 The Key Laboratory of Chemistry for Natural Product of Guizhou Province, Chinese Academy of Science, Guiyang, 550002 China; 5 State Key Laboratory for Conservation and Utilization of Bio-Resources in Yunnan, Center for Life Sciences, School of Life Sciences, Yunnan University, Kunming, 650091 China

**Keywords:** lysosome, alkaloids, lysosomal cell death, STAT3, *Caenorhabditis elegans*

## Abstract

Lysosomes are degradation and signaling centers within the cell, and their dysfunction impairs a wide variety of cellular processes. To understand the cellular effect of lysosome damage, we screened natural small-molecule compounds that induce lysosomal abnormality using *Caenorhabditis elegans* (*C*. *elegans*) as a model system. A group of vobasinyl-ibogan type bisindole alkaloids (ervachinines A–D) were identified that caused lysosome enlargement in *C*. *elegans* macrophage-like cells. Intriguingly, these compounds triggered cell death in the germ line independently of the canonical apoptosis pathway. In mammalian cells, ervachinines A–D induced lysosomal enlargement and damage, leading to leakage of cathepsin proteases, inhibition of autophagosome degradation and necrotic cell death. Further analysis revealed that this ervachinine-induced lysosome damage and lysosomal cell death depended on STAT3 signaling, but not RIP1 or RIP3 signaling. These findings suggest that lysosome-damaging compounds are promising reagents for dissecting signaling mechanisms underlying lysosome homeostasis and lysosome-related human disorders.

**Electronic supplementary material:**

The online version of this article (10.1007/s13238-018-0520-0) contains supplementary material, which is available to authorized users.

## Introduction

Lysosomes are acidic single-membrane organelles that function as the major sites for digesting cargoes received from several pathways, including endocytosis, phagocytosis and autophagy. The lysosome contains >60 different hydrolytic enzymes, many of which are activated at low pH. The acidity of the lysosomal lumen is generated and maintained by v-ATPase, an ATP-dependent proton pump. The lysosome contains over 150 membrane proteins that are required for the integrity and homeostasis of the organelle (Saftig and Klumperman, 2009). Impairment of lysosomal function is responsible for more than 70 lysosomal storage diseases (LSDs) (Macauley, 2016) and contributes to many other human diseases, such as neurodegenerative disorders and cancers (Nixon, 2013; Perera et al., 2015). Lysosomes also participate in several types of cell death including apoptosis and necroptosis (Taniguchi et al., 2015; Kreuzaler et al., 2011). In particular, lysosomal damage leads to lysosomal cell death (LCD) under specific physiological or pathological conditions, for instance, mammary gland involution after lactation, neutrophil aging, and bacterial infection (Sargeant et al., 2014; Loison et al., 2014; Prince et al., 2008).

LCD is characterized by lysosomal membrane permeabilization (LMP) and release of cathepsin proteases into the cytoplasm (Boya and Kroemer, 2008). In the cytoplasm, cathepsins act as executioners of cell death by mechanisms that are not well understood. Interestingly, while LCD is known to occur independently of caspases, cytoplasmic cathepsins can cleave Bid, promoting mitochondrial translocation of the proapoptotic proteins Bax and Bak, which in turn induce mitochondrial membrane permeabilization and caspase-dependent apoptosis (Oberle et al., 2010). Cathepsins can also promote the degradation of antiapoptotic proteins such as XIAP to facilitate apoptosis (Oberle et al., 2010). In addition, cathepsins promote LMP and thus amplify LCD signals (Oberle et al., 2010). The caspase-independent feature of LCD offers an important alternative for designing therapeutic strategies for cancer treatment. Because cancer cells generally carry mutations in proapoptotic factors or overexpression of antiapoptotic factors, they are usually resistant to apoptosis. However, it has been found that LCD can be induced in such apoptosis-resistant cells (Gonzalez et al., 2012). Induction of LCD was also found to restrict propagation of invading bacterial pathogens (Almaguel et al., 2010; Zhu et al., 2015). Thus, identifying potent LCD-inducing compounds may provide valuable reagents both for dissecting mechanisms underlying LCD and for treating lysosome-related human diseases.

In this study, we took advantage of the unique endo-lysosome system in macrophage-like cells in *Caenorhabditis elegans* (*C*. *elegans*) to screen for natural compounds that induced lysosomal abnormality. Our screen identified a group of vobasinyl-ibogan type bisindole alkaloids (ervachinines A–D) that caused abnormal lysosome enlargement in both *C*. *elegans* and mammalian cells. We further found that these lysosome-targeting natural compounds induced LMP and LCD in a STAT3-dependent manner. These findings suggest that ervachinines A–D are promising candidates for dissecting the signals underlying lysosome homeostasis and for developing therapeutic reagents for human disorders resulting from defective apoptosis.

## Results

### Using *C*. *elegans* as a model to screen for natural compounds that induce lysosomal abnormality

**Figure 1:**
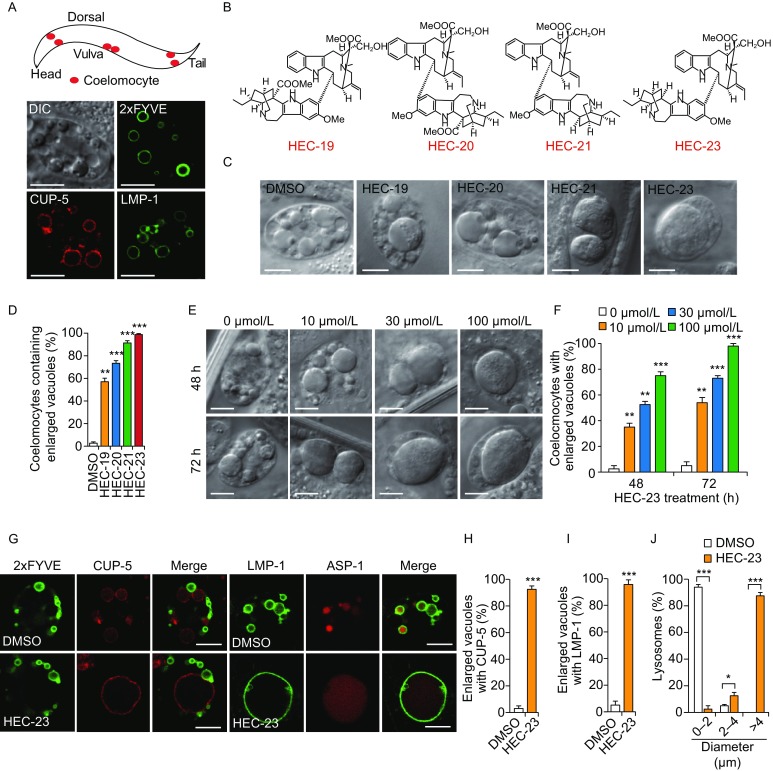
**HEC-23 induces lysosomal enlargement in coelomocytes**. (A) Representative images of endosomes and lysosomes in *C*. *elegans* coelomocytes. The top panel shows a schematic depiction of 3 pairs of coelomocytes (in red) in *C*. *elegans.* The bottom panels show a DIC image of a coelomocyte and images of 2xFYVE::GFP-labeled early endosomes, mCherry::CUP-5-labeled lysosomes, and LMP-1::GFP-labeled lysosomes. Scale bars, 10 μm. (B) Structures of HEC family compounds. (C and D) HEC family compounds induce enlargement of vacuoles in coelomocytes. Worms were treated with indicated HEC compounds at 100 μmol/L for 48 h. DIC images (C) are shown for the vacuoles and quantifications are shown in (D). (E and F) Representative DIC images (E) and quantification (F) of vacuole enlargement induced by HEC-23. (G) Effect of HEC-23 on vacuoles positive for 2xFYVE::GFP, mCherry::CUP-5, LMP-1::GFP and ASP-1::dsRed. Scale bars, 10 μm. (H and I) Quantification of vacuoles labeled with mCherry::CUP-5 (H) and LMP-1::GFP (I) in animals treated with HEC-23. (J) Quantification of lysosome sizes in worms treated with HEC-23 (100 μmol/L, 48 h). Data (mean ± SEM) were from 3 independent experiments. **P < 0.01, ***P < 0.001

*C*. *elegans* has 6 specialized macrophage-like cells, namely coelomocytes, which are highly active in fluid-phase endocytosis (Sato et al., 2016). Coelomocytes contain endosomes and lysosomes that are easily distinguished with differential interference contrast (DIC) optics or fluorescent markers (Fig. 1A). These features make *C*. *elegans* an ideal organism for screening small-molecule compounds that can affect endosome-lysosome trafficking. To identify compounds that induce endosomal or lysosomal abnormalities, we carried out a screen by treating larval stage 4 (L4) worms cultured in liquid medium with individual natural compounds at several concentrations and then observed the change in organelle morphology under DIC optics. A group of bisindole alkaloids isolated from *Ervatamia chinensis* (Meschini et al., 2008; Guo et al., 2012), named as HEC-19 (ervachinine A), HEC-20 (ervachinine C), HEC-21 (ervachinine D) and HEC-23 (ervachinine B), induced vacuolar enlargement in coelomocytes (Fig. 1B–D and Table S1). Among them, HEC-23 had the strongest effect (Fig. 1D), and it induced vacuolar enlargement in time- and dose-dependent manners (Fig. 1E and 1F).

To determine the identities of the enlarged vacuoles induced by HEC-23, we treated worms expressing endosome- or lysosome-specific proteins tagged with fluorescent proteins. HEC-23-enlarged vacuoles were positive for mCherry::CUP-5 (lysosomal calcium channel), LMP-1::GFP (lysosomal membrane protein) and ASP-1::dsRed (lysosomal hydrolase) (Fig. 1G–J). However, HEC-23 did not change the sizes of early endosomes labeled by 2xFYVE::GFP, an indicator of early endosome-specific phosphatidylinositol 3-phosphate (PI3P) (Fig. 1G). These results indicate that HEC-23 specifically enlarged lysosomes in coelomocytes.

### HEC-23 impairs lysosomal degradation and increases the number of cell corpses in the germline

**Figure 2:**
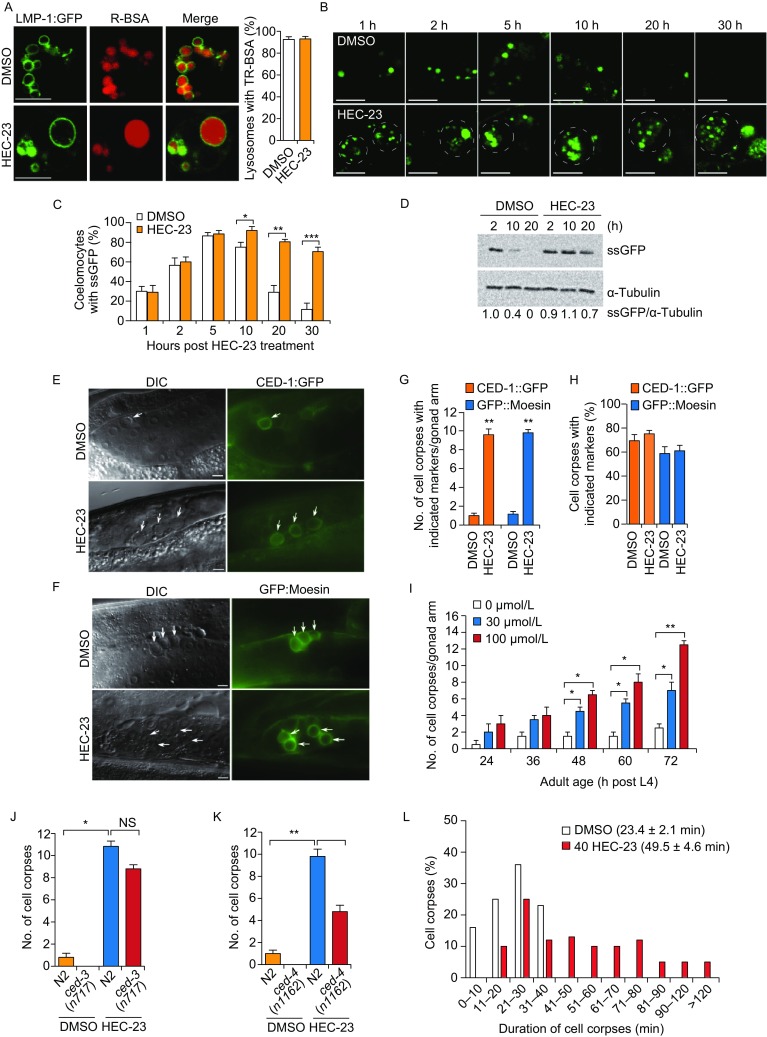
**HEC-23 impairs lysosomal degradation and increases the number of cell corpses in the** ***C***. ***elegans*** **germline**. (A) Representative images of TR-BSA localization in LMP-1::GFP-positive lysosomes following HEC-23 treatment. (B–D) Time-course analysis of ssGFP signals in DMSO- and HEC-23-treated coelomocytes. Expression of ssGFP under the control of a heat-shock promoter was induced at 33°C for 30 min, and the uptake and degradation of ssGFP in coelomocytes were monitored at the indicated time points (B). The dashed circles indicate HEC-23-enlarged lysosomes. Quantifications are shown in (C and D). (E–H) Images (E and F) and quantification (G and H) of HEC-23-induced germ cell corpses labeled with CED-1::GFP (E) or GFP::moesin (F) in the *C*. *elegans* germline. Arrows indicate cell corpses. Scale bars, 20 μm. (I) Quantification of HEC-23-induced germ cell corpses in animals at the indicated adult ages. 30 animals were scored for each time point. (J and K) Quantification of HEC-23-induced germ cell corpses in *ced-3* (I) and *ced-4* (J) loss-of-function mutants. (L) Durations of germ cell corpses in DMSO- and HEC-23 (100 μmol/L)-treated worms. Cell corpses from >30 worms were analyzed. For all quantifications, data (mean ± SEM) were from 3 independent experiments. *P < 0.05, **P < 0.01, ***P < 0.001. NS, not significant

Next, we investigated whether HEC-23 affects the delivery of endocytic cargoes to the lysosome by injecting Texas-Red BSA (TR-BSA) into the body cavity of HEC-23-treated worms and monitoring its appearance in lysosomes in coelomocytes (Liu et al., 2016). Following injection, TR-BSA similarly appeared in lysosomes labeled with LMP-1::GFP in control animals and the enlarged LMP-1::GFP-positive lysosomes in HEC-23-treated animals, suggesting that HEC-23-induced lysosomal enlargement does not affect lysosomal cargo delivery (Fig. 2A). We then used *arIs36* animals to examine whether lysosomal degradation capacity is compromised in the enlarged lysosomes induced by HEC-23. These animals express in the body cavity a secreted soluble GFP (ssGFP) driven by a heat-shock promoter, which is taken up by coelomocytes and degraded in lysosomes (Fares and Greenwald, 2001). We treated *arIs36* animals with HEC-23 and performed time-course monitoring of ssGFP signals in coelomocytes following heat shock. While ssGFP was similarly taken up into coelomocytes in control animals and HEC-23-treated animals, the ssGFP persisted much longer in HEC-23-treated coelomocytes than in control coelomocytes (Fig. 2B–D), indicating that HEC-23-enlarged lysosomes were defective in lysosomal degradation.

Because dysfunction of lysosomes contributes to cell death and affects cell corpse clearance, we tested whether HEC-23 affects apoptosis in germlines. In wild-type animals, HEC-23 treatment caused a significant increase in button-like structures that were encircled by GFP-tagged engulfment receptor CED-1 (CED-1::GFP) or the F-actin-binding protein moesin (GFP::moesin) (Xu et al., 2014) (Fig. 2E–I, and Table S1). This suggests that the button-like structures were dying cells. The *ced-4*(*n1162*) and *ced-3*(*n717*) mutants are respectively deficient in CED-4/Apaf1 and CED-3/Caspase, which are required for apoptosis, HEC-23 also induced button-like cell corpses in these mutants (Fig. 2J, K). Thus, HEC-23 likely induced cell death independently of the canonical apoptosis pathway (Horvitz et al., 1994). These cell corpses persisted much longer than the cell corpses resulting from physiological cell death (Fig. 2L), suggesting that HEC-23-induced lysosome damage also compromised the clearance of cell corpses.

### HEC-23 induces lysosome enlargement in mammalian cells

**Figure 3:**
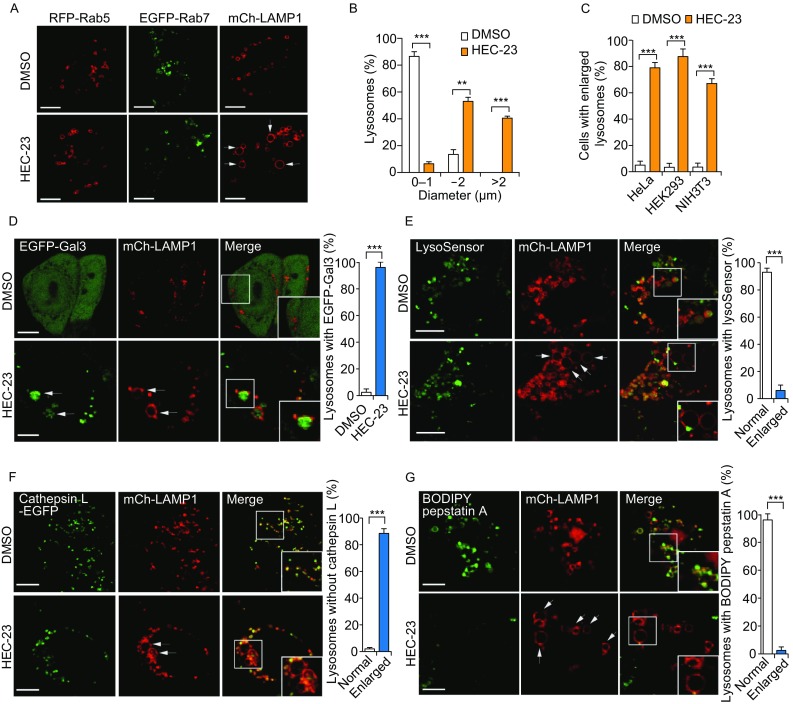
**HEC-23 induces lysosomal damage in mammalian cells**. (A and B) Images (A) and quantification (B) of HEC-23-induced enlargement of LAMP1-positive lysosomes in HeLa cells. (C) Quantification of HEC-23-induced lysosomal enlargement in the indicated cell types. (D) Representative images (left) and quantification (right) of EGFP-Gal3 in mCh-LAMP1-positive lysosomes in HeLa cells treated with HEC-23. (E) Representative images (left) and quantification (right) of LysoSensor Green staining in mCh-LAMP1-positive lysosomes in HeLa cells treated with HEC-23. (F) Representative images (left) and quantification (right) of cathepsin L-EGFP in mCh-LAMP1-positive lysosomes in HeLa cells treated with HEC-23. (G) Representative images (left) and quantification (right) of BODIPY-Pepstatin A in mCh-LAMP1-positive lysosomes in HeLa cells treated with HEC-23. In (D–G), boxed regions in the merged images are magnified and shown in the bottom right corners. Cells were treated with HEC-23 at 10 μmol/L for 3 h. Data (mean ± SEM) were from 3 independent experiments. **P < 0.01, ***P < 0.001. Bars represent 10 μm in all images

Given that HEC-23 impaired lysosomal degradation and induced non-apoptotic cell death in *C*. *elegans*, we investigated whether HEC-23 had a similar role in mammalian cells. In HeLa cells expressing RFP-Rab5, EGFP-Rab7 and mCherry-LAMP1, which label early endosomes, late endosomes and lysosomes, respectively, HEC-23 treatment caused a strong enlargement of LAMP1-positive lysosomes, while no obvious change in Rab5- or Rab7-positive organelles was detected (Fig. 3A). The HEC-23-treated cells contained lysosomes with diameters up to 2 μm, compared with lysosomes in diameter ≤1 μm in control cells (Fig. 3B). HEC-23 similarly enlarged lysosomes in human HEK293 cells and mouse NIH3T3 cells (Fig. 3C). These findings suggested that HEC-23 had a specific effect on lysosomes in mammalian cells. To investigate this further, we examined the integrity of these enlarged lysosomes using GFP-fused Galectin 3 (EGFP-Gal3), which binds to β-galactoside on luminal glycoproteins of endosomes or lysosomes with ruptured membranes (Liu et al., 1995; Ono et al., 2003; Maejima et al., 2013). In mock-treated cells, EGFP-Gal3 was distributed evenly in the cytoplasm; however, EGFP-Gal3 formed punctate structures in the enlarged lysosomes following HEC-23 treatment (Fig. 3D). This suggested that HEC-23 caused damage to lysosomal membranes. To corroborate this, we examined the acidification of lysosomes using LysoSensor staining. While lysosomes in mock-treated cells were positive for LysoSensor Green, no LysoSensor Green staining was detected for the enlarged lysosomes in HEC-23-treated cells (Fig. 3E). Thus, the acidification of HEC-23-enlarged lysosomes was impaired. Furthermore, we examined the localization of cathepsin L, a lysosomal cathepsin protease. In DMSO-treated cells, EGFP-tagged cathepsin L localized well in LAMP1-positive lysosomes, but HEC-23-induced enlarged lysosomes did not contain cathepsin L (Fig. 3F), suggesting that lysosomal damage led to leakage of cathepsins from the enlarged lysosomes. Consistent with these findings, the lysosomes in HEC-23-treated cells were not stained by BODIPY pepstatin A, which labels mature (cathepsin D-positive) lysosomes (Chen et al., 2000) (Fig. 3G). This result indicates that HEC-23 impairs lysosomal maturation.

### HEC-23 inhibits autophagosome degradation

**Figure 4:**
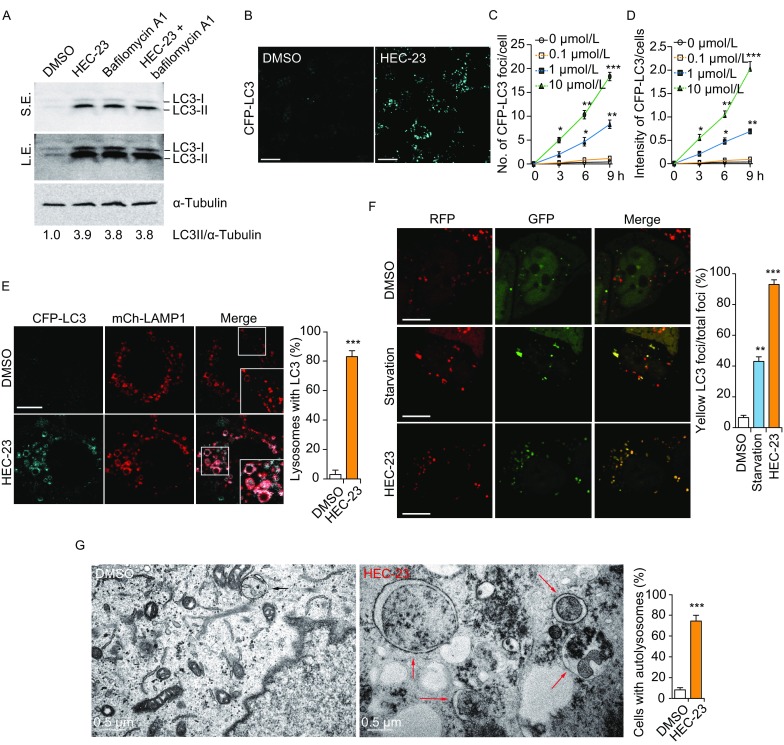
**HEC-23 impairs autophagosome degradation**. (A) Immuno-blotting of LC3 in HeLa cells treated with HEC-23 (10 μmol/L, 6 h) and bafilomycin A1 (0.4 μmol/L, 6 h). Relative fold changes of LC3-II intensity are indicated at the bottom. (B–D) Representative images (B) and quantifications (C and D) of CFP-LC3 foci in HeLa cells treated with HEC-23. (E) Representative images (left) and quantification (right) of the colocalization of stably expressed CFP-LC3 and mCh-LAMP1 in cells treated without or with HEC-23. Boxed regions in the merged images are magnified and shown in the bottom right corners. (F) Representative images of RFP-GFP-LC3 foci in cells treated with DMSO, starvation (EBSS, 3 h), or HEC-23 (10 μmol/L, 3 h). (G) Transmission electron microscopy images of cargo-containing autolysosomes in HeLa cells without or with HEC-23 treatment (10 μmol/L, 6 h). For quantifications, data (mean ± SEM) were from 3 independent experiments. *P < 0.05, **P < 0.01, ***P < 0.001. Bars represent 10 μm in all images except (G)

Because HEC-23 induced lysosome damage, we investigated if HEC-23 has an effect on autophagy, a lysosome-dependent process for degradation of cellular contents. HEC-23 treatment strongly increased the endogenous level of the autophagosome marker LC3II in HeLa cells (Fig. 4A). The increase was similar to that caused by the lysosomal v-ATPase inhibitor bafilomycin A1. Combined treatment with HEC-23 and bafilomycin A1 did not cause a further increase in the LC3 level (Fig. 4A). This result indicated that HEC-23 only impaired lysosomal degradation, rather than inducing autophagosome formation (Fig. 4A). In HeLa cells stably expressing CFP-LC3, HEC-23 induced a strong increase in the number and intensity of CFP-LC3 foci, in contrast to the even distribution of CFP-LC3 in control DMSO-treated cells (Fig. 4B–D). The HEC-23-induced CFP-LC3 foci colocalized with the enlarged lysosomes (Fig. 4E), suggesting that the fusion of autophagosomes with lysosomes was normal but the degradation was compromised. In HeLa cells transiently expressing RFP-GFP-LC3, HEC-23 induced formation of LC3 foci positive for both GFP and RFP (Fig. 4F), indicating that HEC-23 caused accumulation of autophagosomes by inhibiting their degradation. In contrast, starvation-induced LC3 foci were mostly positive for RFP, owing to the quenching of GFP signals following autolysosome formation (Fig. 4F). Using transmission electron microscopy (TEM), we confirmed that there was indeed an increase in the number of autophagosomes/autolysosomes with accumulated substrates in the lumen (Fig. 4G). Thus, HEC-23-induced lysosome damage inhibited degradation of autophagosomes.

### HEC-23-induced lysosomal enlargement depends on STAT3 activation

**Figure 5:**
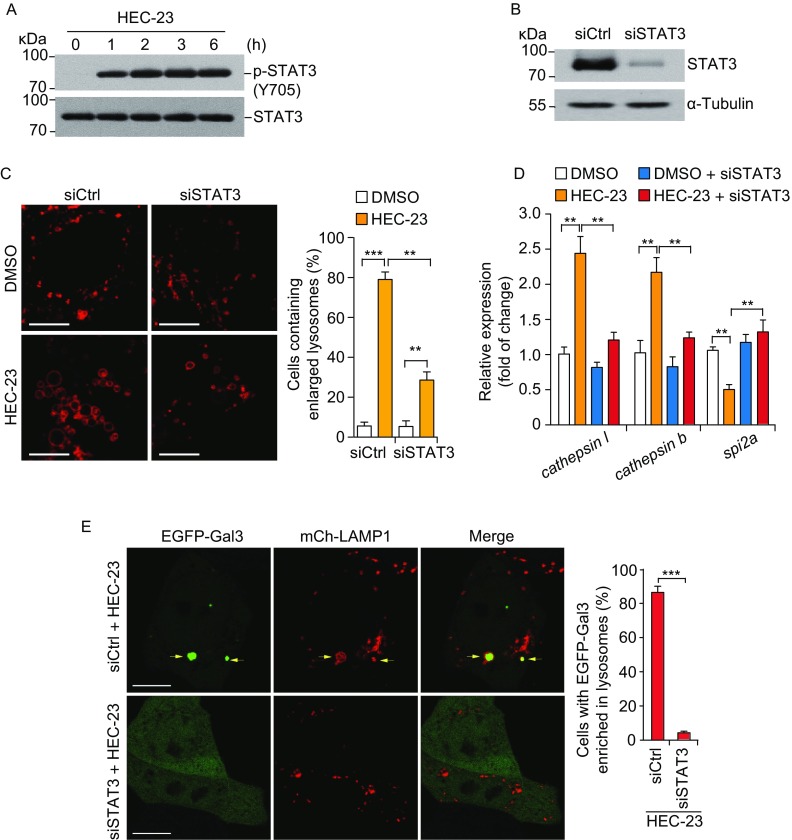
**HEC-23-induced enlargement of lysosomes depends on STAT3 activation**. (A) Immuno-blotting of STAT3 phosphorylation at Y705 in HeLa cells following HEC-23 treatment. (B) Immuno-blotting of STAT3 in HeLa cells treated with siSTAT3. (C) Representative images (left) and quantification (right) of lysosomes labeled with mCherry-LAMP1 in HEC-23-treated HeLa cells pretreated with control siRNA (siCtrl) and STAT3 siRNA (siSTAT3). (D) qPCR-analysis of the expression of STAT3 target genes in siCtrl or siSTAT3 cells treated with HEC-23. (E) Representative images (left) and quantification (right) of lysosome damage in HEC-23-treated HeLa cells pretreated with control siRNA (siCtrl) and STAT3 siRNA (siSTAT3). The concentration of HEC-23 was 10 μmol/L in all treatments. Data representing mean ± SEM were derived from 3 independent experiments. **P < 0.01, ***P < 0.001 (Scale bars, 10 μm)

Recently, it was found that STAT3 controls cell death during mammary gland involution by regulating LMP (Kreuzaler et al., 2011; Sargeant et al., 2014). During this process, STAT3 is activated, which up-regulates expression of cathepsins B and L but down-regulates their endogenous inhibitor Spi2A, causing LMP-mediated necrosis in mammary epithelial cells. We thus investigated if STAT3 signaling is involved in HEC-23-induced lysosomal enlargement. HEC-23 strongly increased the phosphorylation of STAT3 at tyrosine 705 (Y705) (Fig. 5A). Importantly, knocking down *STAT3* significantly suppressed HEC-23-induced lysosomal enlargement and damage (Fig. 5B, 5C and 5E). HEC-23 treatment resulted in elevated expression of *cathepsin l* and *cathepsin b*, but decreased the expression of *spi2a*, all of which are STAT3 target genes (Fig. 5D). Furthermore, knocking down *STAT3* reversed the HEC-23-induced changes in the expression of these genes (Fig. 5D). Altogether, these findings suggest that STAT3 signaling is required for HEC-23-induced lysosomal enlargement and damage.

### HEC-23 induces cathepsin-dependent necrosis through STAT3 signaling

**Figure 6:**
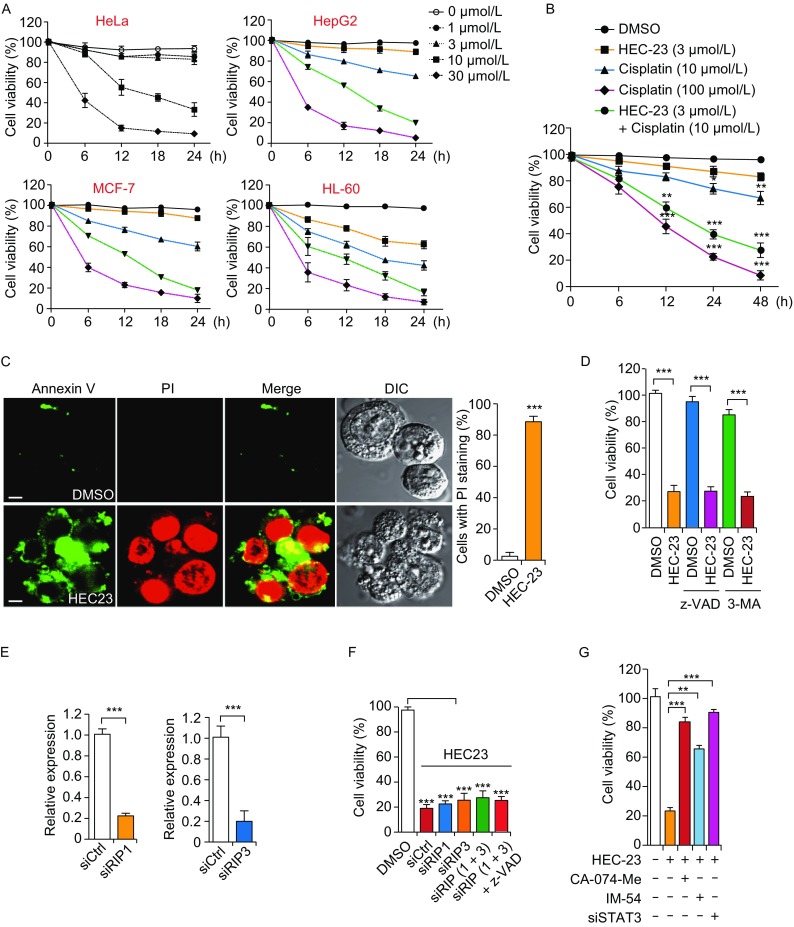
**HEC23-induced cell death is dependent on the STAT3-cathepsin pathway**. (A) Quantifications of cell death induced by HEC-23 in the indicated types of carcinoma cells. (B) Quantification of cell death induced by HEC-23 together with or without cisplatin in HeLa cells. (C) Representative images (left) and flow cytometry quantification (right) of necrosis induced by HEC-23 (10 μmol/L, 12 h). (D) Quantification of cell death induced by HEC-23 (10 μmol/L, 24 h) in HeLa cells pre-treated with z-VAD (20 μmol/L) or 3-MA (1 μmol/L) for 30 min. (E) qPCR evaluation of the knockdown efficiency of siRIP1 and siRIP3. (F) Quantification of the viability of HeLa cells treated with HEC-23 and siRIP1, siRIP3 or both in the presence or absence of z-VAD. (G) Quantification of cell viability in siCtrl or siSTAT3 HeLa cells treated with HEC-23, CA-074-Me or IM-54. HeLa cells were transfected with siRNA for 48 h, followed by treatment with CA-074-Me (20 μmol/L) or IM-54 (2 μmol/L) for 30 min. The cells were then treated with HEC-23 for 24 h. MTT assays were performed to examine the viability of the cells. The concentration of HEC-23 was 10 μmol/L in all treatments. Data representing mean ± SEM were derived from 3 independent experiments. **P < 0.01, ***P < 0.001 (Scale bars, 10 μm)

In carcinoma cells, including HeLa, HepG2, MCF-7 and HL-60, HEC-23 induced cell death in a concentration-dependent manner (Fig. 6A). In addition, HEC-23 had a synergistic effect on cell death with cisplatin, a well-established anti-tumor therapeutic drug (Fruh et al., 2016) (Fig. 6B). Further analysis indicated that >90% of the dying HEC-23-treated cells were positive for propidium iodide (PI) staining, indicating that HEC-23 likely induced non-apoptotic cell death (Fig. 6C). In support of this hypothesis, pretreatment with the pan-caspase inhibitor z-VAD or the autophagic inhibitor 3-MA (3-methyladenine) did not inhibit the cell death induced by HEC-23 (Fig. 6D).

**Figure 7:**
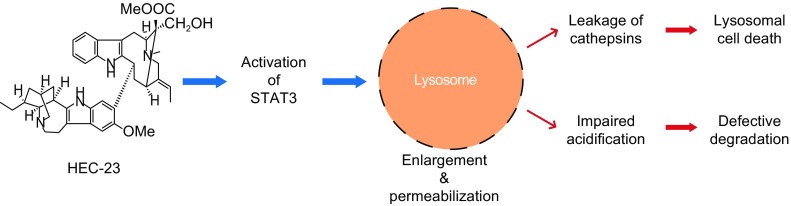
**Schematic diagram of HEC23-induced lysosomal enlargement and cell death**

To determine which signaling pathway is required for HEC-23-induced cell death, we performed siRNA (small interfering RNA) to knock down RIP1 and RIP3, the key regulators of necroptosis (Sun and Wang, 2014). Knockdown of RIP1, RIP3, or both, did not affect HEC-23-induced cell death (Fig. 6E, F), suggesting that HEC-23 does not act through these two factors. In contrast, treatment of cells with *STAT3* siRNA, the cathepsin inhibitor CA-074-Me or the necrosis inhibitor IM-54 strongly suppressed HEC-23-induced cell death (Fig. 6G). These findings indicated that HEC23-induced STAT3 activation not only upregulated gene expression of cathepsins (Fig. 5D), but also resulted in LMP (Fig. 5C, E) and subsequent leakage of cathepsins (Fig. 3F) and necrosis (Fig. 6G). Altogether, these results indicate that STAT3 signaling is required for HEC-23-induced lysosomal dysfunction and LCD (Fig. 7).

## Discussion

In this study, we present *C*. *elegans* as a model for screening natural compounds that target lysosomes. We found that a group of alkaloids, named HECs, enlarge the size and impair the integrity, acidification and digestion capacity of lysosomes, and these effects are conserved in different species. HEC-induced lysosomal impairment results in the accumulation of cell corpses and non-apoptotic cell death in *C*. *elegans* and lysosomal cell death in mammalian cells.

The use of *C*. *elegans* to identify lysosome-targeting small-molecule compounds has many advantages. Firstly, the macrophage-like coelomocytes are active in fluid-phase endocytosis and pinocytosis, which facilitate the entry of compounds into the cell. Secondly, lysosomes are easily identified in a living animal using DIC optics or fluorescently labeled makers. Thirdly, the powerful genetic approaches and the availability of numerous mutant alleles make it possible to dissect the mechanisms underlying the function of the compounds.

Apoptosis and necrosis have been extensively studied during the development of organisms and the pathogenesis of diseases. In the present study, we find that mutation of neither *ced-3* nor *ced-4* could totally block the HEC-23-induced elevation of cell corpses. This demonstrates that HEC-23 induces non-apoptotic cell death in *C*. *elegans*. We also performed knock-down of RIP1 and RIP3, which did not reverse HEC-23-induced necrosis in mammalian cells. These data demonstrate that HEC-23-induced necrosis is independent of the RIP1/3 pathway.

Intriguingly, LMP also plays a crucial physiological role in regression of the mammary gland. Conditional deletion of STAT3 causes an obviously delay in involution of the mammary gland and reduces the level of cell death (Kreuzaler et al., 2011; Sargeant et al., 2014). In the present study, we found that HEC-23 induces the activation of STAT3, up-regulation of cathepsins B and L and down-regulation of Spi2A. Importantly, knock-down of STAT3 abolished lysosomal enlargement and necrosis induced by HEC-23 treatment. Furthermore, inhibition of cathepsins reverses the HEC-23-induced reduction in cell viability. These findings indicate that HEC23-induced necrosis is dependent on the STAT3-cathepsin pathway.

We found that HEC-23 treatment significantly promotes the phosphorylation of STAT3 at Y705. Unfortunately, we failed to obtain functional biotin-labeled compounds related to HEC-23, since they all lost the ability to enlarge lysosomes in cells. However, we can still propose how HEC-23 may interact with potential targets in the JAK-STAT3 signaling pathways. Firstly, HEC-23 may function as an agonist to directly bind with and activate JAK (Janus kinases), which in turn phosphorylates STAT3 at the Y705 site (Vainchenker and Constantinescu, 2013). Secondly, HEC-23 may function as an antagonist to bind with Src homology region 2 domain-containing phosphatase-1 (SHP-1) or suppressor of cytokine signaling (SOCS), which inhibits JAK activity in the cytoplasm (Vainchenker and Constantinescu, 2013). Thirdly, HEC-23 may function as an antagonist to bind with E3 SUMO-protein ligases (PIASs) or protein tyrosine phosphatases (PTPs), which inhibit STAT3 transcription activity in the nucleus (Vainchenker and Constantinescu, 2013).

Usually, LCD remains functional in apoptosis-resistant cancer cells. The fact that HEC-23 induced non-apoptotic cell death in several different cancer cell lines suggests that HEC-23 family compounds can potentially be used to develop therapeutic reagents for cancers that are resistant to apoptosis-based therapy. Notably, the antimalarial agent mefloquine was found to induce LMP and release of cathepsins into the cytoplasm of human acute myeloid leukemia (AML) cells, which provides a novel and promising therapeutic strategy for AML (Sukhai et al., 2013). Further studies are needed to investigate the application of HEC-23 and STAT3 activation to the therapeutic treatment of AML and other cancers.

## Materials and Methods

### *C*. *elegans* strains and genetics

The Bristol strain N2 is used as wild type. The mutant alleles used in this study are *vps-18*(*tm1125*), *arl-8*(*tm2388*), *ced-3*(*n717*) and *ced-4*(*n1162*). The integrated arrays are: *smIs34*(P_*ced-1*_*ced-1*::*gfp*), *yqIs121*(P_*ced-1*_*gfp*::*moesin*), *cdIs85*(P_u*nc-122*_*2xfyve*::*gfp*), *cdIs97*(P_*unc-122*_*mCherry*::*cup-5*), *cdIs131*(P_*unc-122*_*gfp*::*rab-5*), *pwIs50*(P_*lmp-1*_*lmp-1*::*gfp*), and *tmIs225*(P_*asp-1*_*asp-1*::*dsRed*)*. C*. *elegans* cultures and genetic crosses were performed according to standard procedures.

### Screen for natural compounds that induce enlargement of lysosomes

All compounds used in this study were isolated from plants and compound structures were determined by means of 1D NMR, 2D NMR and MS as previously reported (Li et al., 2016). In total, 257 natural compounds (35 alkaloids, 23 triterpenes, 46 diterpenes, 22 sesquiterpenes, 17 monoterpenes, 18 tetranortriterpenoids, 12 flavonoids, 11 coumarins, 25 sterols, 27 lignanoids, 11 saponins, and 10 cyclic peptides) were tested individually for their ability to induce lysosome enlargement. Worms were cultured in M9 solution (1 L contains 3 g KH_2_PO_4_, 6 g Na_2_HPO_4_, 5 g NaCl and 1 mmol/L MgSO_4_) with X1666. 20–30 worms at L4 stage were transferred into liquid culture with or without different natural compounds in 24-well plates in a shaker (120 rpm, 20°C). 48 h or 72 h later, worms were examined for lysosomal changes in coelomocytes under a fluorescence microscope with DIC optics. The investigators were blinded to compound identities during the screen.

### Microscopy and trafficking experiments in coelomocytes

Adult worms were immobilized with 2.5 mmol/L levamisole in M9 solution and mounted on 2% agarose pads for imaging. DIC pictures were captured by using an AxioImager M1 (Carl Zeiss). Fluorescence images were obtained by using an inverted FV1000 confocal microscope system (IX81; Olympus). TR-BSA trafficking assays in *C*. *elegans* coelomocytes were performed as previously described^29^. In brief, TR-BSA (Sigma-Aldrich; 1 mg/mL in water) was injected into the body cavity of adult worms. Then worms were cultured on NGM plates seeded with *Escherichia coli* OP50. Worms were imaged by confocal microscopy at different time points after injection. For each time point, similar results were obtained in more than 30 coelomocytes from 15–20 different worms.

### Germ cell corpse analysis

Animals synchronized to different adult stages were scored for germ cell corpses under Nomarski optics. Animals were grown in liquid culture at 20°C unless otherwise indicated. For cell corpse analysis in HEC-23-treated worms, animals were scored for germ cell corpses after 12 h, 24 h, 36 h, 48 h and 72 h. At each time point, germ cell corpses in the meiotic region of one gonad arm were counted for every animal, and ≥30 animals were analyzed.

### Cell culture, transfection and reagents

All cell lines were cultured at 37°C with 5% CO_2_ in Dulbecco’s modified Eagle’s medium (DMEM) supplemented with 10% fetal bovine serum (FBS) (HyClone), 100 U/mL penicillin and 100 mg/mL streptomycin. No cell lines used in this study were found in the database of commonly misidentified cell lines that is maintained by ICLAC and NCBI Biosample. All cell lines were from American type culture collection (ATCC). Transient transfections were performed with Lipofectamine 2000 (Invitrogen, Carlsbad, CA) following the manufacturers’ instructions. TR-BSA and CA-074-Me were from Tocris Bioscience (Bristol, UK). bafilomycin A1, 3-MA, z-VAD, necrosis inhibitor IM-54 and cisplatin were from Calbiochem (Darmstadt, Germany). The mature lysosome dye BODIPY-pepstatin A, LysoSensor, Annexin V and PI were purchased from Invitrogen Life Technologies (Carlsbad, CA). The antibody against LC3 was from MBL. Antibodies against p-STAT3 and STAT3 were from Cell Signaling Technology. Mouse monoclonal antibodies for α-tubulin were purchased from Sigma-Aldrich (St. Louis, MO). HRP-, Cy3- and FITC-conjugated secondary antibodies were obtained from Jackson ImmunoResearch Laboratories (West Grove, PA).

### Western blot

Cells were lysed in ice-cold RIPA buffer (20 mmol/L Tris-HCl pH 7.5, 100 mmol/L NaCl, 0.1% SDS, 0.5% sodium deoxycholate, 1 mmol/L PMSF) containing Complete Protease Inhibitor Cocktail (1 tablet in 50 mL RIPA buffer) and Phosphatase Inhibitor Cocktail Tablets (1 tablet in 10 mL RIPA buffer) (Roche, Basel, Switzerland). Cell lysates were spun down at 12,000 rpm for 10 min at 4°C. 20 μg of each supernatant were resolved by sodium dodecyl sulfate polyacrylamide gel electrophoresis (SDS-PAGE) and probed with the indicated antibodies. α-Tubulin was used as the internal control.

### Small interfering RNA (siRNA)

RNA oligos used for siRNA in this study were:

**Table:** 

Human gene	Oligo
STAT3	5′-UGUAAUGCAUGACAGCCUGTT-3′ 5′-AUUGUCUUUCUUCUGCCGCTT-3′ 5′-UUGAUGUUGAACCUUCGUCTT-3′
RIP1	5′-AUCCCUGCUCUCUUCAGUGTT-3′ 5′-AGGGCUGCUUUCCUUGGCCTT-3′ 5′-UCAUCAGCCUGGAGUCCAGTT-3′
RIP3	5′-AUUUGAAUGUAAAGGACUCTT-3′ 5′-AGUAACAAAUUCAUGGCACTT-3′ 5′-AUUUCAUACAACAGGACGCTT-3′
Control siRNA	5′-UUCUCCGAACGUGUCACGUTT-3′

Cells were transfected with 100 pmol RNA oligos twice (at 0 h and 24 h) using Lipofectamine 2000 in 6-well plates or confocal culture dishes. The efficiency of siRNAs was evaluated by Western blot or qRT-PCR.

### Quantitative real-time reverse-transcription PCR (qPCR)

RNA was isolated from cells by using TRIzol reagent (Invitrogen) as recommended by the manufacturer. A reverse transcription kit (Promega) was used to reverse transcribe RNA (1 μg) in a 20 μL-reaction mixture. Quantification of gene expression was performed using a real-time PCR system (7900HT Fast; Applied Biosystems) in triplicate. Amplification of the sequence of interest was normalized with the reference endogenous *GAPDH* gene.

### Expression vectors

The mammalian expression vector pEGFP-N2-cathepsin L was constructed by inserting the cDNA of cathepsin L between the *Hin*dIII and *Kpn*I sites of the pEGFP-N2 vector using standard protocols confirmed by sequencing.

The following vectors were kindly provided by other scientists: mCherry-LAMP1 (Dr. Li Yu, Tsinghua University, China), EGFP-Galectin3 (Dr. Tamotsu Yoshimori, Osaka University, Japan), RFP-GFP-LC3 (Dr. Hong Zhang, Institute of Biophysics, CAS). All expression constructs were confirmed by DNA sequencing.

### Transmission electron microscopy

HeLa cells were cultured on plastic cover slices with DMEM containing 10% FBS. After HEC-23 treatment for 3 h, the cells were fixed in fixation buffer (2.5% glutaraldehyde, 1% paraformaldehyde in PBS) on ice for 1 h. The samples were then post-fixed by 1% OsO_4_ for 2 h, followed by dehydration in a graded ethanol series (30%, 50%, 70%, 90% and 100%). After rinsing with propylene oxide (100%) for 3 times, the samples were infiltrated stepwise in increasing concentrations of embed 812 resin (propylene oxide:resin 2:1 for 3 h and 1:1 for 5 h). Then, the samples were incubated in 100% fresh resin twice for 8 h, and transferred into fresh resin in an embedding mold and polymerized in a 60°C oven for 3 days. Ultrathin sections (70 nm) were generated with a diamond knife (Diatome) on an ultramicrotome (Ultracut UCT; Leica Microsystems), and collected on copper grids (EMS). The slices on copper grids were stained with 2% UAc and 1% citric acid for 10 min. Then, the samples were visualized with a JEM-1400 TEM at 80 kV. Pictures were recorded with a Gatan832 (4k × 2.7k) CCD camera.

### MTT assay for cell viability

Cells were cultured in 96-well plates with DMEM containing 10% FBS. After HEC-23 treatment for 24 h, 15 μL of dye solution was added into each well. Then the plates were incubated at 37°C for 2 h in a humidified CO_2_ incubator. 100 μL of stop solution was added to each well, and the absorbance was recorded at 570 nm using a 96-well plate reader. A reference wavelength at 630 nm was used. The MTT kit was purchased from Promega (Cat# G4002).

### Cell death quantification by flow cytometry

After treatment with HEC-23 for 24 h, the cells were harvested and washed with PBS. Cells were stained in binding buffer containing Annexin V (5 μL) and PI (10 μL) for 15–30 min in the dark. After extensive washing, cells were suspended in PBS and transferred into tubes for quantification of cell death by flow cytometry using a FACS AriaII machine (BD Biosciences). Data were analyzed by using FlowJo software (FLOWJO, LLC).

### Statistics and reproducibility

Data were analyzed with Prism (GraphPad Software) to generate curves and bar charts. Statistical analyses were performed using *t*-tests or ANOVA. *P* < 0.05, indicated with *, was considered statistically significant. *P* < 0.01, indicated with **, was considered significant. *P* < 0.001, indicated with ***, was considered extremely significant. *P >* 0.05 was considered not significant (NS).

## Electronic supplementary material

Below is the link to the electronic supplementary material.

Supplementary material 1 (PDF 193 kb)
