# Supplementary material for: C. elegans-based screen identifies lysosome-damaging alkaloids that induce STAT3-dependent lysosomal cell death
Source: Protein Cell. 2018 Apr 2;9(12):1013–26. doi: 10.1007/s13238-018-0520-0 (PMC6251801; doi:10.1007/s13238-018-0520-0)
Supplement: Supplementary file 1 — Supplementary material 1 (PDF 193 kb) [file 13238_2018_520_MOESM1_ESM.pdf]

C. elegans-based screen identifies lysosome-damaging alkaloids that induce STAT3-dependent lysosomal cell death

Yang Li[1,3#*], Yu Zhang[2#], Qiwen Gan[3], Meng Xu[3], Xiao Ding[2], Guihua Tang[2], Jingjing Liang[3], Kai Liu[3], Xuezhao Liu[3], Xin Wang[5], Lingli Guo[2], Zhiyang Gao[3], Xiaojiang Hao[2,4*], and Chonglin Yang[3,5*]

[1]Department of Pharmacology, School of Basic Medical Science; Key Laboratory of Metabolism and Molecular Medicine, the Ministry of Education, Fudan University, Shanghai, 200032, China
[2]State Key Laboratory of Phytochemistry and Plant Resources in Western China, Kunming Institute of Botany, Chinese Academy of Sciences, Kunming 650021, China
[3]State Key Laboratory of Molecular Developmental Biology, Institute of Genetics and Developmental Biology, Chinese Academy of Sciences, No.1 West Beichen Road, Chaoyang District, Beijing 100101, China
[4]The Key Laboratory of Chemistry for Natural Product of Guizhou Province and Chinese Academy of Science, Guiyang 550002, China
[5]State Key Laboratory for Conservation and Utilization of Bio-Resources in Yunnan, Center for Life Sciences, and School of Life Sciences, Yunnan University, Kunming 650091, China

[#]These authors contributed equally to this work.
[*]Correspondence should be addressed to Y.L., C.L.Y. or X.J.H
E-mail: Leading contact oceanyangli@fudan.edu.cn,
clyang@genetics.ac.cn or haoxj@mail.kib.ac.cn

Experiment Section

Extraction and Identification of Natural Compounds—All alkaloids used in this study were isolated from *E. chinensis* by the investigators involved in this research and compound structures were determined by means of 1D and 2D NMR as well as MS.

The extraction and isolation procedures are as follows: the powder of *E. chinensis* (7.5 kg) were extracted three times with EtOH–H_2O (95:5, v/v). The combined extracts were concentrated under reduced pressure, followed by partitioning between EtOAc and 3% tartaric acid. The aqueous phase was adjusted to pH 9–10 with saturated Na_2CO_3 and then extracted with $CHCl_3$ to give crude alkaloids (40.0 g). The crude alkaloid extract was then subjected to a silica gel CC (300–400 mesh) using a petroleum ether–Me_2CO gradient (1:0–0:1) to obtain four major fractions A–D. Fraction B (3.8 g) was further purified by silica gel CC (300–400 mesh) with petroleum ether–Me_2CO–Et_2NH (10:1:0.2–4:1:0.2, v/v), then followed by Sephadex LH-20 (MeOH) to afford ervachinine A (20.0 mg), ervachinine C (25.0 mg), and decarbomethoxyvoacamine (60.0 mg). Fraction D (4.4 g) was further purified by reversed phase chromatography on a C18 column (MeOH–H_2O, 20:80–100:0) to give three subfractions (D1–D3). Subfraction D2 (1.2 g) was subjected to silica gel CC eluted with petroleum ether–EtOAc (10:1–3:1, v/v), then followed by Sephadex LH-20 CC (MeOH) to afford ervachinine B (17.0 mg), and ervachinine D (17.5 mg). Subfraction D3 (1.0 g) was further purified by silica gel eluting with petroleum ether–Me_2CO (8:1–4:1, v/v), and then Sephadex LH-20 ($CHCl_3$–MeOH, 1:1) to yield tabernaecorymbosine A (30.0 mg).

HEC-16 (Tabernaecorymbosine A): ^{1}H-NMR (500 MHz，CD_3OD) δ (ppm): 5.30 (1H, dd, *J* = 13.0, 3.5 Hz, H-3), 3.81 (1H, t, *J* = 9.0 Hz, H-5), 3.43 (2H, dd, *J* = 15.8, 3.5 Hz, H-6), 7.62 (1H, d, *J* = 7.5 Hz, H-9), 3.80 (1H, d, *J* = 11.5 Hz, H-17a), 3.63 (1H, d, *J* = 11.5 Hz, H-17b), 1.64 (3H, d, *J* = 7.5 Hz, Me-18), 5.33 (1H, q, *J* = 7.0 Hz, H-19), 2.88 (1H, d, *J* = 14.0 Hz, H-21a), 3.52 (1H, d, *J* = 14.0 Hz, H-21b), 2.35 (3H, s, Me-COOMe), 2.55 (3H, s, Me-NMe), 7.15 (1H, d, *J* = 8.0 Hz, H-9′), 6.81 (1H, d, *J* = 8.0 Hz, H-10′), 0.81 (3H, t, *J* = 7.5 Hz, H-18′), 1.29 (2H, q, *J* = 7.5, 1.42 Hz, H-19′), 3.36 (1H, s, H-21′), 3.66 (3H, s, Me-COOMe′), 3.95 (3H, s, Me-11′); ^{13}C-NMR (125 MHz, CD_3OD) δ (ppm): 138.8 (C-2), 36.7 (C-3), 61.8 (C-5), 19.0 (C-6), 110.4 (C-7), 130.9 (C-8), 118.9 (C-9), 119.8 (C-10), 122.7 (C-11), 111.1 (C-12), 138.3 (C-13), 35.4 (C-14), 36.3 (C-15), 54.1 (C-16), 70.2 (C-17), 12.3 (C-18), 120.6 (C-19), 138.4 (C-20), 52.9 (C-21), 50.6 (*Me*-COOMe), 175.0 (*CO*-COOMe), 42.6 (C-*NMe*), 136.5 (C-2′), 52.9 (C-3′), 54.2 (C-5′), 22.7 (C-6′), 110.0 (C-7′), 125.7 (C-8′), 117.7 (C-9′), 106.4 (C-10′), 153.3 (C-11′), 116.4 (C-12′), 136.7 (C-13′), 28.6 (C-14′), 33.0 (C-15′), 55.6 (C-16′), 35.7 (C-17′), 11.9 (C-18′), 27.6 (C-19′), 40.0 (C-20′), 57.5 (C-21′), 52.9 (*Me*-COOMe′), 175.9 (*CO*-COOMe′), 57.4 (C-OMe′). ESI-MS: *m/z* 735 $[M + H]^+$.

HEC-19 (Ervachinine A): ^{1}H-NMR (500 MHz，$CDCl_3$) δ (ppm): 5.07 (1H, br d, *J* = 13.0, H-3), 3.99 (1H, t, *J* = 8.0 Hz, H-5), 3.48 (1H, dd, *J* = 13.8, 9.6 Hz, H-6a), 3.26 (1H, dd, *J* = 13.8, 8.0 Hz, H-6b), 7.46 (1H, br d, *J* = 7.5 Hz, H-9), 7.01 (1H, m, H-10), 7.00 (1H, m, H-11), 6.97 (1H, m, H-12), 2.42 (1H, m, H-14a), 1.95 (1H, m, H-14b),

3.43 (1H, m, H-15), 3.69 (1H, d, J = 11.0 Hz, H-17a), 3.46 (1H, d, J = 11.0 Hz, H-17b), 1.58 (3H, d, J = 8.0 Hz, H-18), 5.27 (2H, q, J = 8.0 Hz, H-19), 3.58 (1H, d, J = 11.0 Hz, H-21a), 3.04 (1H, d, J = 11.0 Hz, H-21b), 7.62 (1H, br s, NH), 2.32 (3H, s, OMe), 2.59 (3H, s, NMe), 2.81 (1H, br d, J = 10.0 Hz, H-3′a), 2.64 (1H, br d, J = 10.0 Hz, H-3′b), 3.30 (1H, m, H-5′a), 3.07 (1H, ddd, J = 18.0, 12.0, 6.0 Hz, H-5′b), 3.02 (1H, br d, J = 9.6, 6.0 Hz, H-6′a), 2.90 (1H, br d, J = 9.6, 6.0 Hz, H-6′b), 6.85 (1H, s, H-9′), 6.59 (1H, s, H-12′), 1.69 (1H, m, H-15′a), 1.02 (1H, m, H-15′b), 2.38 (1H, br d, J = 16.5 Hz, H-17′a), 1.66 (1H, br d, J = 16.5 Hz, H-17′b), 0.80 (3H, t, J = 8.0 Hz, H-18′), 1.56 (1H, m, H-19′a), 1.34 (1H, m, H-19′b), 1.19 (1H, m, H-20′), 3.44 (1H, br s, H-21′), 3.91 (3H, s, 10′-OMe), 3.54 (3H, s, OMe). ^{13}C-NMR (125 MHz, $CDCl_3$) δ (ppm): 138.0 (C-2), 37.2 (C-3), 60.0 (C-5), 17.4 (C-6), 109.9 (C-7), 129.8 (C-8), 117.5 (C-9), 118.9 (C-10), 120.4 (C-11), 110.2 (C-12), 136.0 (C-13), 36.4 (C-14), 35.7 (C-15), 52.0 (C-16), 70.3 (C-17), 12.2 (C-18), 121.7 (C-19), 136.1 (C-20), 51.8 (C-21), 50.4 (*Me*-COOMe), 173.9 (*CO*-COOMe), 42.0 (C-*NMe*), 137.2 (C-2′), 51.9 (C-3′), 53.0 (C-5′), 22.1 (C-6′), 110.2 (C-7′), 127.3 (C-8′), 99.1 (C-9′), 150.1 (C-10′), 129.9 (C-11′), 110.4 (C-12′), 130.2 (C-13′), 27.3 (C-14′), 31.9 (C-15′), 54.8 (C-16′), 36.4 (C-17′), 11.6 (C-18′), 26.7 (C-19′), 38.9 (C-20′), 57.0 (C-21′), 52.4 (*Me*-COOMe′), 175.2 (*CO*-COOMe′), 56.0 (C-OMe′). ESI-MS: *m/z* 735 $[M + H]^+$.

HEC-20 (Ervachinine C): ^{1}H-NMR (400 MHz，CD_3OD) δ (ppm): 5.11 (1H, br d, J = 12.8, H-3), 3.74 (1H, t, J = 10.4 Hz, H-5), 3.51 (1H, dd, J = 16.2, 7.2 Hz, H-6a), 3.23 (1H, dd, J = 16.2, 8.4 Hz, H-6b), 7.51 (1H, dd, J = 7.0, 2.5 Hz, H-9), 6.98 (1H, m, H-10), 6.98 (1H, m, H-11), 7.10 (1H, dd, J = 7.2, 2.8 Hz, H-12), 2.48 (1H, m, H-14a), 2.00 (1H, m, H-14b), 3.62 (1H, dd, J = 12.0, 5.6 Hz, H-15), 3.78 (1H, d, J = 10.8 Hz, H-17a), 3.56 (1H, d, J = 10.8 Hz, H-17b), 1.65 (3H, d, J = 6.0 Hz, H-18), 5.29 (2H, q, J = 6.0 Hz, H-19), 3.48 (1H, d, J = 10.0 Hz, H-21a), 2.75 (1H, d, J = 10.0 Hz, H-21b), 2.28 (3H, s, OMe), 2.39 (3H, s, NMe), 3.10 (1H, dd, J = 13.2, 7.2 Hz, H-3′a), 2.81 (1H, dd, J = 13.2, 6.4 Hz, H-3′b), 2.66 (1H, dd, J = 9.6, 7.2 Hz, H-5′a), 2.56 (1H, m, H-5′b), 2.71 (1H, t, J = 7.2 Hz, H-6′a), 2.59 (1H, t, J = 7.2 Hz, H-6′b), 6.70 (1H, s, H-9′), 6.83 (1H, s, H-12′), 1.55 (1H, m, H-14′), 1.59 (1H, m, H-15′a), 0.96 (1H, m, H-15′b), 2.55 (1H, br d, J = 14.0 Hz, H-17′a), 1.71 (1H, br d, J = 14.0 Hz, H-17′b), 0.81 (3H, t, J = 7.2 Hz, H-18′), 1.42 (1H, m, H-19′a), 1.32 (1H, m, H-19′b), 1.24 (1H, m, H-20′), 3.40 (1H, br s, H-21′), 3.92 (3H, s, 11′-OMe), 3.62 (3H, s, OMe).^{13}C-NMR (100 MHz, CD_3OD) δ (ppm): 139.7 (C-2), 39.1 (C-3), 62.1 (C-5), 18.7 (C-6), 111.3 (C-7), 131.3 (C-8), 118.4 (C-9), 119.2 (C-10), 122.0 (C-11), 111.0 (C-12), 138.0 (C-13), 38.6 (C-14), 36.4 (C-15), 54.0 (C-16), 70.3 (C-17), 12.4 (C-18), 120.3 (C-19), 138.7 (C-20), 52.9 (C-21), 50.3 (*Me*-COOMe), 174.6 (*CO*-COOMe), 42.5 (C-*NMe*), 136.7 (C-2′), 54.5 (C-3′), 53.7 (C-5′), 22.7 (C-6′), 110.4 (C-7′), 123.5 (C-8′), 118.3 (C-9′), 128.5 (C-10′), 154.5 (C-11′), 93.9 (C-12′), 136.6 (C-13′), 28.6 (C-14′), 33.0 (C-15′), 56.1 (C-16′), 36.9 (C-17′), 12.1 (C-18′), 27.8 (C-19′), 39.9 (C-20′), 58.0 (C-21′), 52.8 (*Me*-COOMe′), 176.4 (*CO*-COOMe′), 56.2 (C-OMe′). ESI-MS: *m/z* 735 $[M + H]^+$.

HEC-21 (Ervachinine D): ^{1}H-NMR (400 MHz，CD_3OD) δ (ppm): 5.13 (1H, br d, J = 13.0, H-3), 3.78 (1H, t, J = 11.2 Hz, H-5), 3.56 (1H, m, H-6a), 3.26 (1H, dd, J = 14.8, 8.0 Hz, H-6b), 7.53 (1H, br d, J = 7.0 Hz, H-9), 7.00 (1H, m, H-10), 7.00 (1H, m,

H-11), 7.10 (1H, dd, J = 7.2, 2.4 Hz, H-12), 2.51 (1H, m, H-14a), 2.01 (1H, m, H-14b), 3.63 (1H, dd, J = 11.6, 6.8 Hz, H-15), 3.81 (1H, d, J = 12.0 Hz, H-17a), 3.60 (1H, d, J = 12.0 Hz, H-17b), 1.69 (3H, d, J = 6.4 Hz, H-18), 5.36 (2H, q, J = 6.4 Hz, H-19), 3.57 (1H, d, J = 13.2 Hz, H-21a), 2.87 (1H, d, J = 13.2 Hz, H-21b), 2.31 (3H, s, OMe), 2.49 (3H, s, NMe), 2.90 (1H, br d, J = 8.4 Hz, H-3′a), 2.75 (1H, br d, J = 8.4 Hz, H-3′b), 3.04 (1H, br d, J = 13.2 Hz, H-5′a), 2.82 (1H, br d, J = 13.2 Hz, H-5′b), 3.00 (1H, m, H-6′a), 2.20 (1H, m, H-6′b), 6.67 (1H, s, H-9′), 6.83 (1H, s, H-12′), 1.69 (1H, m, H-14′), 1.79 (1H, m, H-15′a), 1.12 (1H, m, H-15′b), 2.89 (1H, br d, J = 8.4 Hz, H-16′), 2.01 (1H, br d, J = 12.4 Hz, H-17′a), 1.48 (1H, br d, J = 12.4 Hz, H-17′b), 0.88 (3H, t, J = 6.8 Hz, H-18′), 1.48 (1H, q, J = 6.8 Hz, H-19′a), 1.42 (1H, q, J = 6.8 Hz, H-19′b), 1.53 (1H, m, H-20′), 2.70 (1H, br s, H-21′), 3.95 (3H, s, 11′-OMe), ^{13}C-NMR (100 MHz, CD_3OD) δ (ppm): 139.9 (C-2), 39.1 (C-3), 62.1 (C-5), 18.7 (C-6), 111.2 (C-7), 131.4 (C-8), 118.2 (C-9), 119.2 (C-10), 122.0 (C-11), 111.0 (C-12), 138.0 (C-13), 38.7 (C-14), 36.4 (C-15), 54.1 (C-16), 70.3 (C-17), 12.3 (C-18), 120.2 (C-19), 138.9 (C-20), 53.1 (C-21), 50.4 (*Me*-COOMe), 174.5 (*CO*-COOMe), 42.6 (C-*NMe*), 141.5 (C-2′), 50.6 (C-3′), 55.4 (C-5′), 21.3 (C-6′), 108.6 (C-7′), 124.7 (C-8′), 118.1 (C-9′), 128.2 (C-10′), 154.0 (C-11′), 93.7 (C-12′), 135.6 (C-13′), 27.7 (C-14′), 33.0 (C-15′), 41.9 (C-16′), 35.3 (C-17′), 12.3 (C-18′), 28.5 (C-19′), 43.3 (C-20′), 59.4 (C-21′), 56.3 (C-OMe′). ESI-MS: *m/z* 677 $[M + H]^+$.

HEC-22 (Decarbomethoxyvoacamine): ^{13}C-NMR (150 MHz，CD_3OD) δ (ppm): 139.3 (C-2), 39.5 (C-3), 61.3 (C-5), 20.7 (C-6), 111.2 (C-7), 131.2 (C-8), 118.3 (C-9), 120.3 (C-10), 122.2 (C-11), 110.9 (C-12), 138.0 (C-13), 38.3 (C-14), 33.4 (C-15), 48.2 (C-16), 12.6 (C-18), 119.5 (C-19), 139.3 (C-20), 53.4 (C-21), 50.0 (Me-COOMe), 172.5 (*CO*-COOMe), 42.4 (C-*NMe*), 143.2 (C-2′), 49.7 (C-3′), 55.9 (C-5′), 21.6 (C-6′), 108.7 (C-7′), 129.4 (C-8′), 99.7 (C-9′), 152.2 (C-10′), 129.3 (C-11′), 111.2 (C-12′), 130.2 (C-13′), 27.6 (C-14′), 32.8 (C-15′), 41.7 (C-16′), 35.1 (C-17′), 12.1 (C-18′), 28.7 (C-19′), 42.4 (C-20′), 59.7 (C-21′), 56.6 (C-OMe′). ESI-MS: *m/z* 647 $[M + H]^+$.

HEC-23 (Ervachinine B): ^{1}H-NMR (400 MHz，$CDCl_3$) δ (ppm): 5.13 (1H, br d, J = 13.0, H-3), 3.91 (1H, t, J = 9.0 Hz, H-5), 3.52 (1H, dd, J = 15.0, 9.0 Hz, H-6a), 3.27 (1H, dd, J = 15.0, 6.6 Hz, H-6b), 7.55 (1H, br d, J = 6.0 Hz, H-9), 7.06 (1H, m, H-10), 7.05 (1H, m, H-11), 7.08 (1H, br d, J = 6.6, H-12), 2.49 (1H, m, H-14a), 2.02 (1H, ddd, J = 9.6, 6.6, 2.4, H-14b), 3.51 (1H, t, J = 9.6, H-15), 3.71 (2H, t, J = 12.0 Hz, H-17), 1.66 (3H, d, J = 6.0 Hz, H-18), 5.37 (2H, q, J = 6.0 Hz, H-19), 3.57 (1H, d, J = 12.0 Hz, H-21a), 2.94 (1H, d, J = 12.0 Hz, H-21b), 7.67 (1H, br s, NH), 2.40 (3H, s, OMe), 2.55 (3H, s, NMe), 3.01 (1H, br d, J = 9.0 Hz, H-3′a), 2.95 (1H, br d, J = 9.0 Hz, H-3′b), 3.35 (1H, m, H-5′a), 3.08 (1H, dt, J = 13.2, 4.2 Hz, H-5′b), 3.30 (1H, t, J = 4.2 Hz, H-6′a), 2.59 (1H, br d, J = 13.2 Hz, H-6′b), 6.92 (1H, s, H-9′), 6.62 (1H, s, H-12′), 1.79 (1H, m, H-14′), 1.75 (1H, br t, J = 11.4, H-15′a), 1.16 (1H, m, H-15′b), 2.77 (1H, m, H-16′), 1.94 (1H, br t, J = 12.0 Hz, H-17′a), 1.50 (1H, m, H-17′b), 0.86 (3H, t, J = 6.6 Hz, H-18′), 1.50 (1H, m, H-19′a), 1.42 (1H, m, H-19′b), 1.50 (1H, m, H-20′), 2.76 (1H, br s, H-21′), 3.98 (3H, s, 10′-OMe). ^{13}C-NMR (100 MHz, $CDCl_3$) δ (ppm): 138.2 (C-2), 37.3 (C-3), 60.1 (C-5), 17.2 (C-6), 110.5 (C-7), 129.9 (C-8), 117.5 (C-9), 118.9 (C-10), 121.7 (C-11), 110.0 (C-12), 136.1 (C-13), 36.6 (C-14), 36.0 (C-15), 52.0 (C-16), 70.7 (C-17), 12.2 (C-18), 119.9 (C-19), 136.7 (C-20), 52.0

(C-21), 50.3 (*Me*-COOMe), 174.0 (*CO*-COOMe), 42.0 (C-*NMe*), 136.8 (C-2′), 49.9 (C-3′), 54.2 (C-5′), 20.7 (C-6′), 108.9 (C-7′), 128.3 (C-8′), 98.6 (C-9′), 150.9 (C-10′), 128.8 (C-11′), 110.3 (C-12′), 129.2 (C-13′), 26.4 (C-14′), 32.0 (C-15′), 41.3 (C-16′), 34.1 (C-17′), 11.9 (C-18′), 27.8 (C-19′), 41.9 (C-20′), 57.7 (C-21′), 55.9 (C-OMe′). ESI-MS: *m/z* 677 $[M + H]^+$.

Supplementary Table 1. **HEC compounds induced lysosome enlargement and cell death in both *C. elegans* and mammalian cells.**

Compounds	Name (CAS number)	Structure	% of coelomocytes with enlarged lysosomes	No. of cell corpses per gonad arm	% of HeLa cells with enlarged lysosomes	% of cell death in total cells
HEC-16	Tabernaecorymbosine A (1262306-81-9)	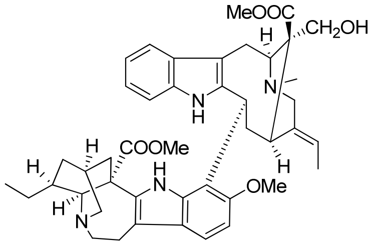	1.8	1.0	3.1	1.6
HEC-19	Ervachinine A (1346017-27-3)	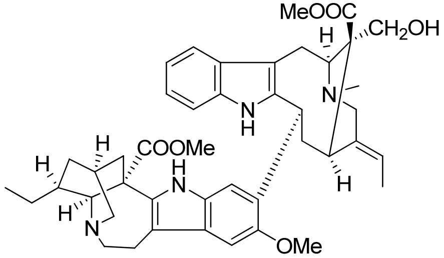	58.6	1.9	32.9	30.6
HEC-20	Ervachinine C (1346017-29-5)	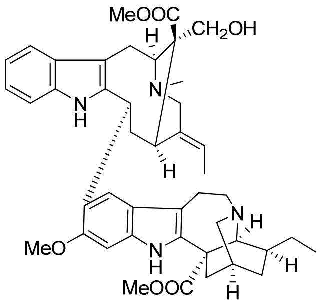	72.5	2.3	55.3	51.8

HEC-21	Ervachinine D (1346017-30-8)	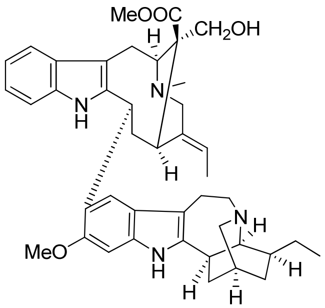	90.3	8.3	70.5	72.3
HEC-22	Decarbomethoxyvoacamine (30890-52-9)	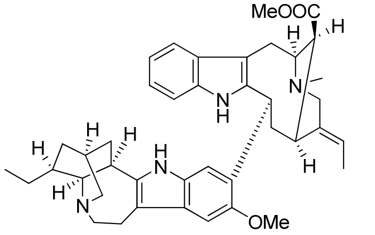	1.5	1.2	3.8	2.1
HEC-23	Ervachinine B (1346017-28-4)	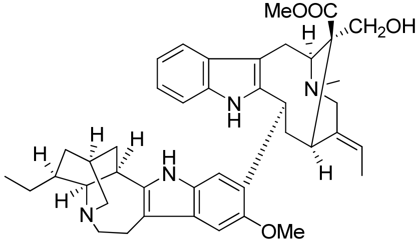	97.2	9.5	78.6	83.5
DMSO			0.0	1.2	2.2	0.5

(>25 worms or >100 cells per group were scored.)
